# Supplementary material for: Responses of Tomato Photosystem II Photochemistry to Pegylated Zinc-Doped Ferrite Nanoparticles
Source: Nanomaterials (Basel). 2025 Feb 13;15(4):288. doi: 10.3390/nano15040288 (PMC11858530; doi:10.3390/nano15040288)
Supplement: Supplementary file 1 [file nanomaterials-15-00288-s001.zip › nanomaterials-3424877-supplementary.pdf]

nanomaterials

Article

Responses of Tomato Photosystem II Photochemistry to Pegylated Zinc-doped Ferrite Nanoparticles

Ilektra Sperdouli, Kleoniki Giannousi, Julietta Moustaka, Orestis Antonoglou, Catherine Dendrinou-Samara and Michael Moustakas

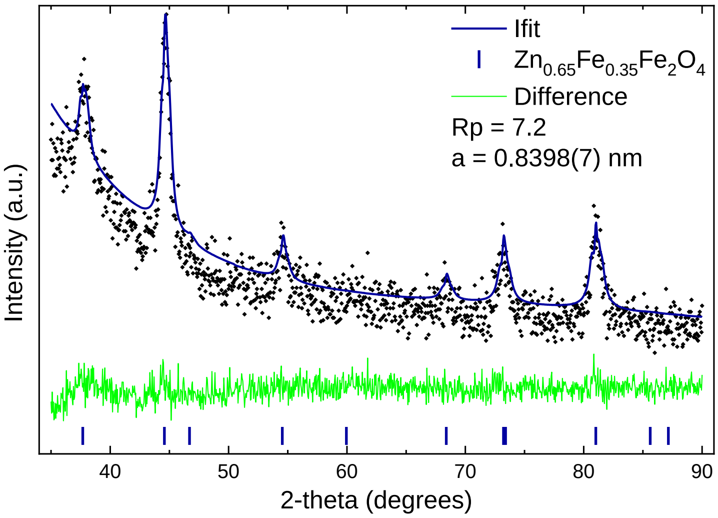

Figure S1. The output from the Rietveld refinement analysis of the XRD pattern of the sample. The plus signs show raw experimental data, and the continuous line overlapping them refers to the calculated data. The vertical bars are the expected Bragg reflection positions. The difference between the experimental data and the calculated data is shown at the bottom.

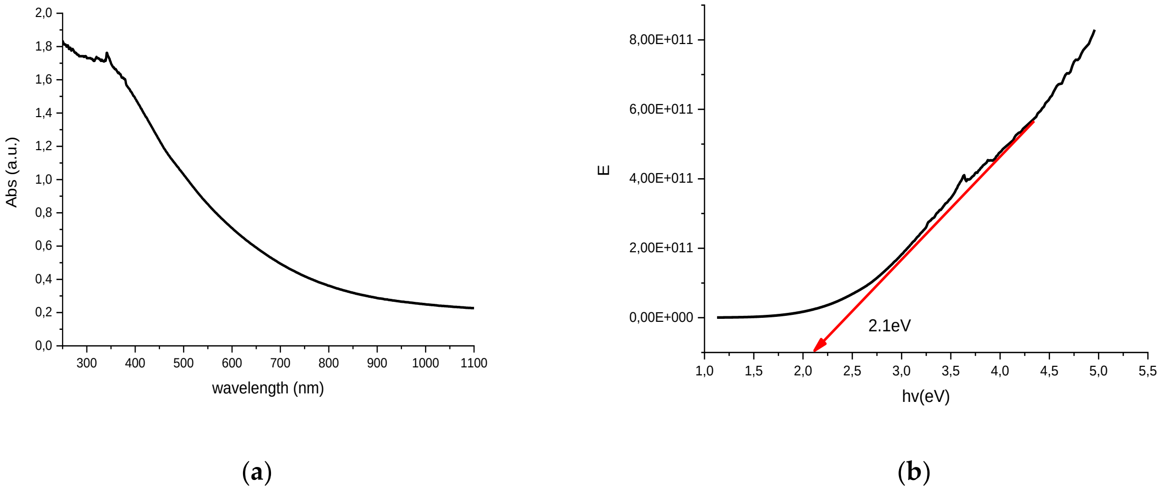

Figure S2. UV-Vis spectrum of ZnFer NPs **(a)**. Tauc Plot diagram and calculation of band gap energy of ZnFer NPs **(b)**.

Table S1. Definitions of the chlorophyll fluorescence parameters

Parameter	Definition	Calculation
F_o	Minimum chlorophyll *a* fluorescence in the dark-adapted leaf (PSII centers open)	Obtained by applying measuring photon irradiance of 1.2 μmol photons m^{-2} s^{-1}
F_m	Maximum chlorophyll *a* fluorescence in the dark-adapted leaf (PSII centers closed)	Obtained with a saturating pulse (SP) of 6000 μmol photons m^{-2} s^{-1}
F_o'	Minimum chlorophyll *a* fluorescence in the light-adapted leaf	It was computed by the Imaging Win software V2.41a (Heinz Walz GmbH, Effeltrich, Germany) as $Fo' = Fo/(Fv/Fm + Fo/Fm')$
F_m'	Maximum chlorophyll *a* fluorescence in the light-adapted leaf	Measured with saturating pulses (SPs) every 20 s for 5 min after application of the actinic light (AL) of 426 μmol photons m^{-2} s^{-1} or 1000 μmol photons m^{-2} s^{-1}
F_s	Steady-state photosynthesis	Measured after 5 min illumination time before switching off the actinic light (AL) of 426 μmol photons m^{-2} s^{-1} or 1000 μmol photons m^{-2} s^{-1}
Fv/Fo	Efficiency of the oxygen evolving complex (OEC) on the donor side of PSII	$(Fm - Fo)/Fo$
Fv/Fm	Maximum efficiency of PSII photochemistry	$(Fm - Fo)/Fm$
Φ_{PSII}	Effective quantum yield of PSII photochemistry	$(Fm' - Fs)/Fm'$
Φ_{NPQ}	Quantum yield of regulated non-photochemical energy loss in PSII	$Fs/Fm' - Fs/Fm$
Φ_{NO}	Quantum yield of non-regulated energy loss in PSII	Fs/Fm
Fv'/Fm'	Efficiency of the open PSII reaction centers	$(Fm' - Fo')/Fm'$
ETR	Electron transport rate	Φ_{PSII} × PAR × c × abs, where PAR is the photosynthetically active radiation, c is 0.5, and abs is the total light absorption of the leaf taken as 0.84
qp	Photochemical quenching, representing the redox state of quinone A (Q_A), or in other words the fraction of open PSII reaction centers based on the "puddle" model for the photosynthetic unit	$(Fm' - Fs)/(Fm' - Fo')$
NPQ	Non-photochemical quenching reflecting the dissipation of excitation energy as heat	$(Fm - Fm')/Fm'$
EXC	Excess excitation energy	$(1 - q_p)/Fv'/Fm'$
1-qL	The fraction of closed PSII reaction centres based on the "lake" model for the photosynthetic unit	$1- (q_p \times F_o'/F_s)$
